# The climate and nature crisis: implications for cancer control

**DOI:** 10.1093/jncics/pkad091

**Published:** 2023-11-23

**Authors:** Leticia M Nogueira

**Affiliations:** Surveillance and Health Equity Science, American Cancer Society, Atlanta, GA, USA

The editorial published in several health journals this month by Abbasi et al. (1) accurately describes the indivisible crisis of climate change and biodiversity loss as a global health emergency, highlighting the usual suspects (eg, infections, water-borne diseases, extreme temperatures). Even if we dismiss the value of biodiverse environments in providing a blueprint for the development of several pharmaceutical agents, including cancer treatment drugs (2), the joint crisis of climate change and biodiversity loss has immediate and urgent implications for cancer control.

Climate change alters the frequency and behavior of extreme weather events, complicating disaster preparedness and response as communities face increasingly unpredictable circumstances, which can lead to disruptions in access to cancer care and worse outcomes (3,4). Further, the physical, psychological, and socioeconomic consequences of cancer diagnosis and treatment can increase individuals’ vulnerability to climate hazards (5). For example, exposure to a wildfire among patients recovering from lung cancer surgery is associated with worse overall survival (6).

Among the many shared causes and connections between climate change and biodiversity loss, the industrialization of the food system has the most relevant implications to cancer control. Current food production relies heavily on land clearing, making industrialized food production the primary driver of biodiversity loss (7). Further, each step of the industrialized food system also emits greenhouse gases, which are driving climate change, including emissions from machinery and carbon release during land clearing and tillage, emissions from production and run-off of fertilizers and pesticides used in crop production, emissions from transportation, processing, packaging, food waste, and crop residue (8).

Importantly, the crisis of climate change and biodiversity loss does not have detrimental health consequences only to people who rely on wild species for food and medicine, as highlighted in the editorial in this issue of the Journal (1). Instead, the current industrialized production of food poses health threats to all people, regardless of territory of residence or cultural practices. For example, industrialized agriculture requires large quantities of pesticides (9) compared with the more complex pest-control strategies employed in biodynamic polycultures (10). Pesticide exposure is associated with several detrimental health consequences (11), including increased cancer risk (12), and is increasingly hard to avoid (13).

Contrary to its stated goal, food security and nutritional well-being have worsened globally with increasing industrialization of the food system (14). Further, current support of industrialized food production not only limits the availability and affordability of diverse and nutritious food (15) but also promotes consumption of unhealthy foods (16). For example, policies aimed at artificially reducing the price of fodder and prioritizing food items with longer shelf life encourage consumption of processed meat, an established carcinogen (17).

Therefore, recognizing how the climate and biodiversity crisis hinders cancer control efforts—and acting on available solutions—are integral components of oncologists’ mission. In addition to modifying individual dietary behaviors, oncology professionals can champion more environmentally responsible food procurement and waste management practices at the institutions with which we are affiliated. Further, as trusted sources of information, oncology professionals are well positioned to support policies with public health and environmental co-benefits, such as improved regulatory oversight of pesticide use. We can no longer ignore the detrimental consequences of the climate and biodiversity crisis to cancer control: The time to act is now. Identify the actions available to you today. This is truly a public health emergency.

## Data Availability

No data sources or analyses were used in this editorial.
